# Time to ‘re-think’ physical activity promotion for young people? Results from a repeated cross-sectional study

**DOI:** 10.1186/s12889-017-4136-8

**Published:** 2017-02-17

**Authors:** Paul Best, Mark A. Tully, Rekesh Corepal, Frank Kee, Ruth F. Hunter

**Affiliations:** 1 School of Social Sciences, Education and Social Work, Queen’s University Belfast, Belfast, UK BT7 1NN; 2 Centre for Public Health, Queen’s University Belfast, Belfast, UK BT7 1NN; 3 UKCRC Centre of Excellence for Public Health (NI), Queen’s University Belfast, Belfast, UK BT7 1NN

**Keywords:** UK physical activity guidelines, Knowledge, Health education, Health communication, Young people

## Abstract

**Background:**

The aim of this study was to investigate the relationship between knowledge of the current UK physical activity (PA) guidelines and amount of daily PA using a sample population of 11–16 year olds in Northern Ireland.

**Methods:**

Cross-sectional survey data from the 2010 and 2013 Young Persons’ Behaviour and Attitudes Survey of 10,790 young people provided information on PA, knowledge of guidelines and socio-demographic characteristics. Multinomial logistic regression was used to investigate the associations between knowledge and amount of daily PA.

**Results:**

Results from 2013 showed 67.0% of respondents were aware of PA guidelines with 15.4% reporting meeting them. Males were more likely to meet PA guidelines than females (OR 3.36, 95% CI 2.47, 4.59). Males who were active for 60 min or more, 7 days per week were less likely to be aware of guidelines (OR = 1.51, 95% CI 1.02, 2.24). For females, knowledge of PA guidelines had no significant association with amount of daily PA (OR = 1.74, 95% CI 0.99, 3.07). Those who did not enjoy being active were less likely to meet the guidelines (OR = 0.05, 95% CI 0.02, 0.12).

**Conclusions:**

Knowledge did not appear to be an important predictor of PA in young people. Consequently, threshold based messaging containing recommended minimum PA guideline information may not be appropriate for this age group. Re-branding PA promotion to include the use of humour may offer a new direction for public health messaging based around fun and enjoyment.

## Background

Physical inactivity is the fourth leading cause of mortality in adults, attributable to an estimated 3.2 million deaths worldwide each year [1, 2]. This problem has its origins early in the life course with research demonstrating that the average time spent in physical activity (PA) falls by 60–70% from childhood to adolescence [3, 4]. Physical activity behaviours and habits formed during this time track into adulthood - emphasising the importance of regular PA from a young age [4–6]. The World Health Organisation, recommends children and adolescents should undertake at least 60 min of moderate to vigorous intensity PA each day [7]. Based on self-report data, (in which respondents typically overestimate PA) one review suggested that only 30–40% of young people (<18 years old) are sufficiently active to meet these guidelines [8]. The most rapid period of decline occurs during middle adolescence [4, 9] and as such more attention is being placed upon increasing PA within this age group.

Mass public health communication campaigns are commonly used by Government and public health organisations to increase public knowledge of how much PA is required for health benefits. This is done in order to improve attitudes and intentions, which may lead to better informed choices and subsequent changes in PA behaviour [10]. These approaches are seen to be more cost effective and provide greater reach than individually focused, practitioner led interventions and can be seen in numerous PA campaigns, such as VERB (U.S), Live long Kids (Canada) Change4Life’ (UK), Push Play (New Zealand) ‘Get a Life, Get Active’ and ‘It all adds up’ (Northern Ireland) [11–15]. The effectiveness of PA interventions using mass marketing campaigns to increase knowledge and change PA behaviour has shown some promise within both child and adult populations [12, 16, 17]. Their popularity is spurred by studies showing poor levels of knowledge of PA guidelines among young people and their parents as a possible associated factor for low PA [18, 19].

Informed by core psychological constructs (i.e. knowledge, attitudes and intention) implicit within a number of common behaviour change models e.g. Hierarchy of Effects, Theory of Planned Behaviour etc. [20–23] this type of public health intervention hypothesizes that knowledge can influence attitude, thus leading to changes in behaviour [20, 22] Therefore increasing public knowledge of health behaviours through mass media campaigns is viewed as an attractive population based intervention. However, this ‘one size fits all approach’ is often criticised in favour of more targeted approaches aimed at higher risk sub-populations [24, 25]. Furthermore, many of these campaigns rely heavily upon threshold messages as a means of encouraging behaviour change e.g. “10,000 steps Ghent” [26]. Threshold messages are designed to “implore individuals to attain a specified volume of behaviour” [27] (p. 195) and are often centred upon a particular set of recommendations. While these messages provide a benchmark in which to measure progress, the extent to which they encourage behaviour change is unclear [28]. Brawley and Latimer (2007, p. 171) note that “guidelines tell people what to do, but not why or how they should do it” [24]. Moreover, the majority of theoretical and empirical evidence has been accumulated from adult populations [28]. Little consideration has been given to the applicably of this approach to affect change in younger populations. This is particularly important given the innate physiological and psychological differences across the life course, such as rational thinking, decision making and future orientation [29].

Current research in adult populations [25, 30] has drawn heavily upon evidence and theory to explain the importance of knowledge of PA guidelines as a means of influencing PA behaviour. Therefore, this study investigates the relationship between knowledge of the current UK PA guidelines and the likelihood of meeting them using a sample population of 11–16 year olds in Northern Ireland. Representative cross-sectional data from two distinct time points enabled identification of common correlates of PA behaviour. This has the potential to inform future public health campaigns and how they might be targeted and tailored for young people.

## Methods

Data were collected from the 2010 and 2013 waves of the Young Persons’ Behaviour and Attitudes Survey (YPBAS) [31], a large school based repeated cross-sectional survey of young people aged 11–16 in Northern Ireland (NI) carried out by the NI Statistics and Research Agency (NISRA).

A stratified random sampling procedure, based on census data [32], was used to generate a representative sample based on school size, educational status, management group (i.e. Controlled, Voluntary etc.) and Education and Library Board area (geographical location) across the 175 schools. Data were collected from 77 schools (*n* = 7616 respondents) in 2010 and 75 schools (*n* = 7076 respondents) in 2013. Note that YPBAS contained two separate questionnaires (version A and version B) with pupil’s completing version A or B only. In 2010, both versions included demographic information as well as information on physical activity and other health behaviours. However in 2013 the physical activity items were moved to version B only. As such, relevant data was only available for 3174 respondents in 2013. One class was randomly sampled within each year group across Years 8–12 (aged 11–16) using both self-completed paper based questionnaires (2010) and online questionnaires (2013). In 2010, the response rate across both versions was 88–90% whereas in 2013 it was 85–86%.

Using data from two distinct points increases the reliability and validity of results. The repeated cross-sectional design also enables one to follow group changes over time. These surveys are representative of school size, educational status and geographical locale in NI. Further to this, using two time points enabled the research team to investigate the possible impact of the well-publicised UK Chief Medical Officers’ Report on PA in 2011 [33].

### Dependent variable

#### Physical activity behaviour

Self-reported PA behaviour was measured using the question “over last 7 days, on how many days have you played any sport, done any physical activity, or played actively that made you out of breath or hot and sweaty for a total of at least 60 min each day?”. This single item question was thought to be appropriate for this age group given the differing age ranges involved and mixed ability of participants. Single item assessments of PA have been shown to be an appropriate measure within previous studies with children [34] albeit with different populations. Furthermore, in other PA studies, single item-measures have shown utility when sample sizes are sufficiently large or that additional measures would add to respondent burden [35]. Responses were categorised as: (1) Active 7 days per week for 60 min; (2) Active 5–6 days per week for 60 min (5–6 (3) Active 2–4 days per week for 60 min; (4) Inactive (1 day or less per week). These categories were chosen in recognition of the fact that those who did some but not enough PA, could be very different from those that do no activity at all.

### Predictor variables

#### Knowledge of PA guidelines

Participants were asked “How many minutes do you think you SHOULD spend each day playing sport, doing physical activity or playing actively to make you out of breath or hot or sweaty in order to be healthy?” Responses included 15 min, 30 min, 60 min, 90 min, more than 90 min or ‘don’t know’. Individuals were categorised into two groups; (1) ‘Aware’ (i.e. 60 min) and (2) ‘Unaware’.

#### Socio-demographic factors

Data were collected for gender, age, education status, socio-economic position (SEP) and long standing illness. Age was categorised into three groups “11–12 years old”, “13–14 years old” and “15–16 years old”. Education status was categorised as “secondary” (suggestive of lower to mixed ability) or “grammar” (suggestive of higher ability). Individual SEP was based on provision of free school meals e.g. “Is the pupil entitled to free school meals” (Yes/No). Geographical area based SEP was derived from the Northern Ireland Multiple Deprivation Measure (MDM) [36]. The MDM calculates deprivation using seven different domains (e.g. income, employment, health, education, proximity to services, living environment and crime & disorder) within pre-defined geographical areas across NI. Respondents are divided into quintiles ranging from the most deprived to the least deprived area of residence. Long standing illness was established by the item “Do you have any physical or mental health conditions or illnesses, lasting or expected to last, for 12 months or more?” (Yes/No).

#### Other health related factors

In addition to knowledge of PA guidelines, other variables showing an association with PA were considered based on previous literature [37]. These included enjoyment of sport (*“Do you enjoy sport or physical activity”*), knowledge of other health guidelines (*“How many portions of fruit/vegetables do you think you SHOULD eat each day?”*), fruit and vegetable intake (*“How many portions of fruit/vegetables do you usually eat each day?”*). Enjoyment of sport was categorised as “Yes, a lot”, “Yes, a little” and “No, not at all”. Knowledge of other health guidelines was established by asking respondents about their knowledge of recommended daily fruit and vegetable intake. Individuals were then categorised into two groups; (1) Aware and (2) Unaware. The final item was self-reported fruit and veg intake which was categorised as “none/little” (0–1 portion per day), “some” (2–4 portions per day) and “sufficient” (5 or more portions per day).

### Statistical analyses

Descriptive statistics were calculated using frequencies and percentages for categorical variables, and weighted to reflect age (year group) and gender across NI. Variables were initially included if they had an association at the *p* < 0.10 level. A series of multinomial logistic regression analyses were conducted on both the 2010 and 2013 datasets. Variables were subsequently removed if there were no significant univariate correlation (*p* < 0.05). Self-reported PA behaviour was the dependent variable with those who reported being active for 1 day or less per week (i.e. “Inactive”) assigned as the reference category This approach was chosen as the proportional odds assumption was not met within an ordinal regression model (*p* = 0.01). Separate analyses were also conducted with male and female only samples. Data were analysed using the Statistical Package for Science (SPSS) version 22.0 Software for Windows (SPSS Inc, Chicago, USA).

## Results

### Demographic characteristics

Table 1 shows the demographic characteristics of the 2010 and 2013 YPBAS populations. NI census data shows that 51% of the population are female with 49% male. YPBAS (2010) had a 51% male and 49% female spread whereas YPBAS (2013) had 56% male and 44% female spread. In order to reflect the composition of the Northern Ireland post-primary population, weights were applied to the data to compensate for nonresponse bias in the achieved YPBAS sample. Figures from the 2013/14 School Census were used to derive weights. Key demographic characteristics, namely age, gender, education status, and SEP (area and individual level), were similar for 2010 and 2013 samples, and representative of the NI population.

**Table 1:** Demographic Characteristics of 2010 & 2013 YPBAS Cohorts

Characteristics	2010 N (%)	2013 N (%)
Daily PA		
Active 7 days per week for 60 mins;	788 (12.0%)	462 (15.4%)
Active 5–6 days per week for 60 mins	2335 (35.7%)	648 (21.6%)
Active 2–4 days per week for 60 mins;	2419 (37.0%)	1395 (46.4%)
Inactive 1 day or less per week	1002 (15.3%)	499 (16.6%)
Knowledge of PA Guidelines		
Unaware	2454 (32.6%)	1039 (33.0%)
Aware	5063 (66.5%)	2114 (67.0%)
Gender		
Male	3879 (51.0%)	1613 (50.9%)
Female	3734 (49.0%)	1554 (49.1%)
Age		
11–12 years old	1755 (23.1%)	658 (20.7%)
13–14 years old	3053 (40.3%)	1245 (39.2%)
15–16 years old	2773 (36.6%)	1271 (40.1%)
Education Status		
Grammar	2956 (38.8%)	1125 (36.0%)
Secondary	4660 (61.2%)	1998 (64.0%)
Proportion Free School Meal		
Yes	1390 (18.3%)	546 (18.5%)
No	6209 (81.7%)	2413 (81.5%)
MDM (Quintile)		
1 (most deprived)	1470 (19.8%)	488 (16.4%)
2	1604 (21.6%)	587 (19.8%)
3	1621 (21.8%)	818 (27.6%)
4	1429 (19.3%)	558 (18.8%)
5 (least deprived)	1299 (17.5%)	518 (17.4%)
Long term illness		
Yes	840 (11.2%)	405 (12.9%)
No	6648 (88.8%)	2722 (87.1%)
Do you enjoy PA		
Yes, a lot	2477 (62.7%)	2059 (65.0%)
Yes, a little	1036 (26.2%)	890 (28.1%)
No, not at all	435 (11.0%)	216 (6.8%)
Daily Fruit and Veg Intake		
None/Little (0–1)	1365 (18.3%)	159 (5.1%)
Some (2–4)	5110 (68.4%)	2488 (79.4%)
Sufficient (5 or more)	998 (13.4%)	487 (15.5%)
Knowledge of Fruit and Veg Guidelines		
Unaware	1974 (27.0%)	970 (31.4%)
Aware	5348 (73.0%)	2121 (68.6%)

Weights applied to descriptive data above

*PA* physical Activity

### Reported daily PA and knowledge of PA guidelines

From 2010 (*n* = 785) to 2013 (*n* = 462), there was a marginal increase in self-report participation in at least 60 min PA per day from 12.0 to 15.4%. The number of respondents who correctly identified the current minimum PA guidance (i.e. knowledge of PA guidelines) also marginally increased from 66.5% (*n* = 1360) in 2010 to 67.0% (*n* = 1215) in 2013.

### Correlates of PA behaviour

Tables 2 and 3 show the results of the multinomial logistic regression investigating correlates of PA behaviour among young people in 2010 and 2013.

**Table 2:** Correlates of daily PA behaviour (2010)

Predictor Variable		Active 2–4 days per week	Active 5–6 days per week	Active 7 days per week
		OR [95% CI]	OR [95% CI]	OR [95% CI]
Enjoyment of Sport or PA	No, not at all	0.16 [0.15, 0.23]	0.06 [0.03, 0.09]	0.02 [0.01, 0.07]
	Yes, a little	0.48 [0.37, 0.61]	0.19 [0.14, 0.25]	0.10 [0.06, 0.16]
	Yes, a lot	[Ref.]	–	–
Gender	Male	0.98 [0.78, 1.23]	1.52 [1.20, 1.31]	2.12 [1.55, 2.91]
	Female	[Ref.]	–	–
Age	11–12 years	1.26 [0.93, 1.72]	1.82 [1.32, 2.52]	4.04 [2.66, 6.15]
	13–14 years	1.05 [0.83, 1.33]	1.53 [1.18, 1.98]	2.27 [1.57, 3.28]
	15–16 years	[Ref.]	–	–
Knowledge of PA guidelines	Unaware	0.78 [0.62, 0.98]	0.70 [0.55, 0.89]	1.41 [1.04, 1.93]
	Aware	[Ref.]	–	–
Daily Fruit and Veg Intake	None/little (0–1)	0.52 [0.34, 0.81]	0.15 [0.09, 0.24]	0.12 [0.07, 0.21]
	Some (2–4)	1.06 [0.71, 1.59]	0.45 [0.30, 0.68]	0.25 [0.16, 0.40]
	Sufficient (5 or more)	[Ref.]	–	–

Reference Category: Somewhat Inactive (0–1 days per week)

*PA* physical Acitivity

**Table 3:** Correlates of daily PA behaviour (2013)

Predictor Variable		Active 2–4 days per week	Active 5–6 days per week	Active 7 days per week
		OR [95% CI]	OR [95% CI]	OR [95% CI]
Enjoyment of Sport or PA	No, not at all	0.13 [0.09, 0.20]	0.02 [0.01, 0.05]	0.05 [0.02, 0.12]
	Yes, a little	0.44 [0.35, 0.56]	0.11 [0.08, 0.15]	0.08 [0.05, 0.12]
	Yes, a lot	[Ref.]	*–*	–
Gender	Male	1.23 [0.98, 1.53]	2.43 [1.85, 3.19]	3.36 [2.47, 4.59]
	Female	[Ref.]	*–*	–
Age	11–12 years	1.05 [0.78, 1.42]	1.22 [0.86, 1.74]	1.92 [1.30, 2.83]
	13–14 years	1.33 [1.04, 1.70]	1.66 [1.23, 2.23]	2.12 [1.51, 2.97]
	15–16 years	[Ref.]	–	–
Knowledge of PA guidelines	Unaware	1.00 [0.79, 1.27]	0.91 [0.68, 1.21]	1.74 [1.29, 2.35]
	Aware	[Ref.]	–	–
Daily Fruit and Veg Intake	None/little (0–1)	0.41 [0.27, 0.63]	0.12 [0.07, 0.20]	0.10 [0.06, 0.17]
	Some (2–4)	0.76 [0.51, 1.12]	0.41 [0.27, 0.62]	0.24 [0.15, 0.36]
	Sufficient (5 or more)	[Ref.]	–	–

Reference Category: Somewhat Inactive (0–1 days per week)

In 2010 those achieving the minimum daily PA recommendations (7 days per week for 60 min) were more likely (OR = 1.41, 95% CI 1.04, 1.93) to be unaware of the PA guidelines than those who were inactive. Those who did not meet the guidelines were more likely to be aware of them, for example; *active 5–6 days per week* (OR = 0.70, 95% CI 0.55, 0.89) and *active 2–4 days per week* (OR = 0. 78, 95% CI 0.62, 0.98). In 2013, those achieving the minimum daily PA (7 days) targets were also more likely to be unaware (OR = 1.74, 95% CI 1.29, 2.35). Those who were *active 5–6 days* (OR = 0.91, 95% CI 0.68, 1.21) and those *active 2–4 days* (OR = 1.05, 95% CI 0.79, 1.27) were marginally more likely to be aware of minimum PA requirements however these results were not significant (*p* > 0.05).

### Other Correlates of PA behaviour

Daily fruit and vegetable intake also emerged as an significant correlate, with those who reported eating 2 or more portions a day more likely to meet the minimum PA guidelines (2010: OR = 0.25, 95% CI 0.16, 0.40; 2013: OR = 0.24, 95% CI 0.15, 0.36). Enjoyment of sport or PA was associated with PA behaviour. Those who did not enjoy being active were less likely to meet the guidelines in both 2010 (OR = 0.02, 95% CI 0.01, 0.07) and in 2013 (OR = 0.05, 95% CI 0.02, 0.12).

### Differences in daily PA and knowledge of PA guidelines in males and females

Gender and age were found to be the two most significant factors related to PA behaviour. Chi-square tests for independence showed that in 2010, females (69.0%, *n* = 1360) were significantly (χ2, 75.55, *p* = 0.01) more likely to be aware of the PA guidelines than males (59.9%, *n* = 1215). While knowledge of PA guidelines increased slightly in 2013 for both sexes, females (73.3%, *n* = 1132) remained more knowledgeable than males (38.9%, *n* = 979) (χ2, 46.30, *p* = 0.01).

This trend however was reversed when examining the participants’ reported level of PA. In 2010, 7.2% (*n* = 115) of female participants reported being active for a minimum of 60 min, seven days per week. Within the same period, 14.0% (*n* = 235) of male participants reported being active for a minimum of 60 min, seven days per week (χ2, 22.113, *p* = 0.01). This gender disparity increased further in 2013 with only 8.0% (*n* = 116) of females reporting PA in line with the minimum UK guidelines compared with 20.1% (*n* = 310) of males (χ2, 186.22, *p* = 0.01).

Separate multinomial regression analyses on male and female sub-groups were performed (see Tables 4 and 5). Males were two to three times more likely to report being active for 7 days per week than females in 2010 (OR = 2.12, 95% CI 1.54, 2.91) and 2013 (OR 3.36, 95% CI 2.47, 4.59). For males, correlates of PA behaviour remained the same in 2010 and 2013, i.e. enjoyment of sport or PA; age; knowledge of PA guidelines; daily fruit and vegetable intake (*p* < 0.05). For females, the correlates of PA behaviour consistent across 2010 and 2013 datasets were enjoyment of sport or PA; age and daily fruit and vegetable intake. Males who were meeting daily PA recommendations were less likely to be aware of the PA guidelines in 2010 or 2013 (OR = 1.51, 95% CI 1.02, 2.24). Knowledge of PA guidelines was not a significant correlate with female group in the 2010 or 2013 (*p* > 0.05).

**Table 4:** Correlates of daily PA behaviour, Females Only (2013)

Predictor Variable		Active 2–4 days per week	Active 5–6 days per week	Active 7 days per week
		OR [95% CI]	OR [95% CI]	OR [95% CI]
Enjoyment of Sport or PA	No, not at all	0.18 [0.11, 0.31]	0.01 [0.00, 0.10]	0.05 [0.01, 0.22]
	Yes, a little	0.54 [0.39, 0.73]	0.13 [0.08, 0.21]	0.12 [0.06, 0.22]
	Yes, a lot	[Ref.]	–	–
Age	11–12 years	1.19 [0.80, 1.78]	2.02 [1.17, 3.50]	2.06 [1.05, 4.00]
	13–14 years	1.49 [1.07, 2.06]	2.16 [1.34, 3.50]	2.24 [1.24, 4.07]
	15–16 years	[Ref.]	–	–
Daily Fruit and Veg Intake	None/little (0–1)	0.38 [0.21, 0.67]	0.06 [0.03, 0.15]	0.07 [0.03, 0.16]
	Some (2–4)	0.72 [0.43, 1.21]	0.31 [0.17, 0.57]	0.15 [0.08, 0.29]
	Sufficient (5 or more)	[Ref.]	–	–

Reference Category: Somewhat Inactive (0–1 days per week)

**Table 5:** Correlates of daily PA behaviour, Males Only (2013)

Predictor Variable		Active 2–4 days per week	Active 5–6 days per week	Active 7 days per week
		OR [95% CI]	OR [95% CI]	OR [95% CI]
Enjoyment of Sport or PA	No, not at all	0.07 [0.03, 0.14]	0.01 [0.00, 0.06]	0.04 [0.01, 0.12]
	Yes, a little	0.36 [0.25, 0.52]	0.09 [0.06, 0.15]	0.05 [0.03, 0.10]
	Yes, a lot	[Ref.]	*–*	*–*
Age	11–12 years	1.01 [0.64, 1.60]	0.94 [0.56, 1.55]	1.79 [1.07, 3.02]
	13–14 years	1.14 [0.78, 1.66]	1.32 [0.87, 2.00]	1.80 [1.15, 2.81]
	15–16 years	[Ref.]	–	–
Knowledge of PA guidelines	Unaware	0.83 [0.58, 1.17]	0.74 [0.50, 1.09]	1.51 [1.02, 2.24]
	Aware	[Ref.]	–	–
Daily Fruit and Veg Intake	None/little (0–1)	0.48 [0.25, 0.89]	0.18 [0.09, 0.36]	0.14 [0.07, 0.28]
	Some (2–4)	0.87 [0.48, 1.55]	0.58 [0.31, 1.05]	0.34 [0.19, 0.63]
	Sufficient (5 or more)	[Ref.]	–	–

Reference Category: Somewhat Inactive (0–1 days per week)

## Discussion

Results demonstrated that knowledge of the current PA guidelines does not increase the likelihood of meeting them using a sample population of 11–16 year olds in Northern Ireland, confirmed across two different cohorts. Results showed that a high proportion of respondents were aware of the minimum amount of PA needed to maintain a healthy lifestyle. However, contrary to underpinning theoretical models, knowledge did not appear to be associated with the proportion of young people achieving the minimum amount of daily PA. Separate analyses on male and female sub-groups showed that males, in particular, who are meeting current PA guidelines are less likely to know them.

These results appear counter-intuitive given the prominence of various behaviour change theories with which knowledge is a key component. However, other studies of young people’s PA have shown a similar disconnect between awareness and compliance which partially supports the findings of this paper [38]. One important area to consider is *attitude formation* as a segue between increased knowledge and behaviour change. How information contained in public health campaigns is assimilated and assigned importance may depend largely upon how it is communicated, particularly amongst young people. Results reported here suggest campaigns would do well to include approaches and processes through which PA can be viewed as more enjoyable. One study by Martins et al. [39] found that for many adolescents, having ‘fun’ was a key reason for being active. Physical activity that was over competitive, non-diversified and non-autonomous were seen as barriers. One approach to making PA appear more enjoyable is the use of humour within public health campaigns. This has already gained some momentum with campaigns such as ‘Stoptober’ and ‘the 10 min shake up’ [15, 40]. The ‘Laugh Model’, is a communication framework which emphasises the importance of humour in health promotion messages as opposed to more threshold based information Lister et al. [41]. This is supported by evidence suggesting that young people were more likely to remember humorous health messages [42]. With this in mind, one must consider if current campaigns are viewed by many young people as representing a ‘less enjoyable’ side of PA by presenting it within a framework of rules, objectives and guidelines. The ‘this girl can’ campaign by Sport England is another current example of a mass media campaign that focuses on PA as a social activity which is fun and enjoyable.

While knowledge did not appear to be related to PA, it is important to note that this is not the only factor related to PA behaviour change [3]. More comprehensive socio-ecological approaches that consider personal, cultural and environmental mechanisms are needed [43]. Campaigns that focus on increasing knowledge are very much targeted at personal level changes without considering that one also needs an environment that supports change. Nonetheless, the underlying assumptions behind many traditional public health campaigns are to focus on increasing knowledge as forerunner to behaviour change. This is also in spite of the fact that much of the evidence base has been garnered from adult populations with little consideration given to the differing cognitive processes through which information is received, processed and acted upon by young people. Furthermore, the theoretical underpinnings of these interventions are not without their critics, particularly when applied to young people. In a discussion of Theory of Planned Behaviour (TPB), Sniehotta and colleagues [44] draw attention to TPB’s reliance on ‘rational reasoning’ - a cognitive process which is (arguably) underdeveloped within younger populations. One must therefore consider the validity of some of the core theoretical underpinnings (i.e. knowledge influences intention and behaviour) within these campaigns to affect behaviour change in younger populations. Future public health campaigns may wish to consider alternative theoretical approaches to understanding how best to affect change in younger populations.

In addition to concerns regarding the importance of increasing knowledge of PA guidelines as a behaviour change strategy, there is sometimes a tendency within public health campaigns to adopt a ‘one size fits all’ approach. In a review of nutritional knowledge and behaviour outcomes, Worsley notes ‘lack of relevance’ as a key factor, citing that “knowledge of cholesterol may be more relevant to 60 years olds than to 16 years olds – so why teach it to children?” [45]. In terms of PA this might focus more on gender differences e.g. different framing of messages aimed at males and females via social media channels. There is a risk that individuals may find it difficult to relate to information offered about population parameters suggesting that the more effective campaigns are those that target specific groups [46].

Results shown here have implications for future targeted health promotion campaigns aimed specifically at young people by highlighting the importance of enjoyment in relation to PA behaviour.

### Strengths and limitations

This repeated cross-sectional nature of the survey at two time points enabled confirmation of our findings with two large representative population cohorts. However, there are several limitations through which the findings from this study should be interpreted. Due to the cross-sectional nature of the surveys, causality cannot be inferred. Moreover, as the study used self-report PA measures it may be subject to reporting or social desirability bias [47]. There is also small possibility that the same respondents may have taken part at both time points due to the sampling strategy employed. This however, is believed to be unlikely as the majority of respondents would have been too old to take part again. Due to the limitation in the dataset we were unable to account for clustering at the school level in our analyses. This study also concentrated mainly on individual level factors. Further research should include social and environmental correlates to further our understanding of multi-level population level factors influencing PA and knowledge.

## Conclusion

Physical activity public health campaigns are at risk of becoming over reliant on threshold based messaging to increase knowledge at the expense of other approaches which may be suited to younger populations. Results have shown that increasing knowledge of PA guidelines was not associated with increased PA behaviour in a large, representative sample of young people. This evidence challenges the current paradigm that suggests that increased knowledge will lead to action. Public health campaigns have the difficult task of providing health information to masses (one size fits all) while targeting specific at risk populations (tailoring). As such, threshold messages have become a simple and direct means in which both to measure and inform the public. Whilst knowledge is an important factor relating to health behaviour change in adults, different approaches may be required to ‘re-think’ and engage younger audiences. As such, the validity of some of the core theoretical underpinnings (i.e. that knowledge strongly influences intention and behaviour) within these campaigns to affect behaviour change in younger populations must be re-considered. The significance of enjoyment of PA in relation to levels of PA point to new directions in public health messaging around humour and fun.

## Abbreviations

NI: Northern Ireland
NISRA: Northern Ireland statistics and research agency
PA: Physical activity
TPB: Theory of planned behaviour
UK: United Kingdom
YPBAS: Young persons’ behaviour and attitudes survey
